# Association Between Glymphatic Function and White Matter Microstructural Injury in Patients With Cushing's Disease

**DOI:** 10.1002/brb3.71285

**Published:** 2026-02-28

**Authors:** Yuxiang Sun, Junpeng Xu, Hailong Liu, Qijia Wu, Zihao Zhu, Xiaoteng Yu, Yanyang Zhang

**Affiliations:** ^1^ Department of Neurosurgery Affiliated Hospital of Hebei University Baoding Hebei China; ^2^ Department of Neurosurgery the First Medical Center of Chinese PLA General Hospital Beijing China; ^3^ Department of Neurosurgery, Neuromedicine Center Beijing Shijitan Hospital, Capital Medical University Beijing China; ^4^ Department of Radiotherapy Beijing Tiantan Hospital, Capital Medical University Beijing China; ^5^ Department of Urology, Peking University First Hospital, Institute of Urology Peking University, National Urological Cancer Center Beijing China

**Keywords:** Cushing's disease, DTI‐ALPS, glymphatic function, neuroimaging, white matter damage

## Abstract

**Objective:**

Patients with Cushing's disease (CD) exhibit white matter (WM) microstructural injury, yet the underlying mechanisms remain incompletely understood. This study aims to investigate the role of glymphatic function in WM damage among patients with CD.

**Methods:**

A total of 69 patients with CD and 64 healthy controls(HC) were enrolled. Glymphatic system function was evaluated using the diffusion tensor image analysis along the perivascular space (DTI‐ALPS) index. WM microstructural injury across 42 tracts was assessed via fractional anisotropy (FA) and mean diffusivity (MD). Serum cortisol levels were quantified using chemiluminescence immunoassay.

**Results:**

Compared with HC, patients with CD exhibited significantly reduced DTI‐ALPS indices (*p* = 0.026). Widespread WM microstructural injury was observed, characterized by decreased FA in 25 tracts and increased MD in 40 tracts. Correlation analyses revealed that in patients with CD, the DTI‐ALPS index showed a significant positive correlation with FA values of the right superior longitudinal fasciculus III (SLF III_R; *r* = 0.42, *p* = 0.033) and a significant negative correlation with 00:00 cortisol levels (*r* = −0.354, *p* = 0.004). Furthermore, mediation analysis confirmed that the DTI‐ALPS index fully mediated the effect of 00:00 cortisol level on the reduction of FA in the SLF III_R (ACME = −0.138, 95% CI: [−0.319, −0.013], *p* = 0.021).

**Conclusion:**

This study provides the first evidence of glymphatic functional impairment in patients with CD. Our findings suggest that aberrantly elevated midnight cortisol levels serve as a primary driver of this glymphatic functional impairment. Furthermore, this impaired glymphatic function fully mediates the impact of 00:00 cortisol levels on microstructural injury to the SLF III_R.

## Graphical abstract

This study suggests that glymphatic dysfunction is associated with white matter injury in Cushing's disease. Mediation analysis indicates that impaired glymphatic function may serve as a pathway linking elevated midnight cortisol to structural damage in the right superior longitudinal fasciculus III.

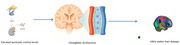

## 1 Introduction

Cushing's disease (CD) is an endogenous hypercortisolism condition caused by the excessive secretion of adrenocorticotropic hormone (ACTH) from a pituitary adenoma (Fleseriu et al. 2021). Existing neuroimaging evidence indicates that patients with CD exhibit widespread microstructural damage to white matter (WM) (van der Werff et al. 2014), with particularly pronounced alterations observed in regions such as the splenium of the corpus callosum, the inferior fronto‐occipital fasciculus, the inferior longitudinal fasciculus, the superior longitudinal fasciculus (SLF), the uncinate fasciculus, and the arcuate fasciculus (Cui et al. 2021). Moreover, these WM microstructural impairments are closely associated with cognitive decline in CD patients (Cui et al. 2021). However, to date, the underlying mechanisms driving WM microstructural damage in individuals with CD remain inadequately elucidated (Xu et al. 2024; Chen et al. 2020).

Recently, the glymphatic system has garnered significant attention for its role in maintaining the homeostasis of brain WM microstructure (Wang et al. 2023; Sabayan and Westendorp 2021). Currently, diffusion tensor imaging analysis along the perivascular spaces (DTI‐ALPS index) is commonly employed as a noninvasive imaging biomarker to reflect the clearance function of the glymphatic system (Taoka et al. 2017). It is generally accepted that a lower DTI‐ALPS index indicates more severe impairment of glymphatic function (Taoka et al. 2017; Park et al. 2023). Simultaneously, fractional anisotropy (FA) and mean diffusivity (MD) are established metrics for assessing WM microstructural damage (Alexander et al. 2007). It is generally understood that a reduction in FA values typically indicates axonal injury or demyelination (Alexander et al. 2007; Budde et al. 2009), whereas an increase in MD values reflects the expansion of the extracellular space or disruption of tissue microstructure (Alexander et al. 2007; Kuwahara et al. 2001). Qin et al. (2023) identified a significant association between a decreased DTI‐ALPS index and both reduced FA values and increased MD values of the corticospinal tract (CST) in patients with ischemic stroke (IS). Similarly, Carotenuto et al. (2022) observed a significant correlation between a lower DTI‐ALPS index and decreased FA values alongside elevated MD values in the WM of individuals with multiple sclerosis (MS). These studies indicate a close association between impaired glymphatic function and damage to WM microstructure (Sabayan and Westendorp 2021). Although the mechanism by which glymphatic dysfunction leads to WM microstructural injury has been established in many neurological disorders (Carlstrom et al. 2022), the functional status of the glymphatic system in patients with CD and its potential relationship with WM microstructure remain unexplored.

Therefore, this study aims to employ DTI‐ALPS indexes to evaluate, for the first time, the functional status of the glymphatic system in patients with CD. Building upon this, we intend to further investigate the intrinsic relationship between the DTI‐ALPS index and microstructural damage indicators (FA and MD values) across 42 whole‐brain WM tracts. This research aspires to elucidate the potential coupling between glymphatic function and WM microstructure in CD patients, thereby offering novel insights into the mechanisms underlying WM microstructural damage in CD.

## 2 Methods

### 2.1 Study Approval and Patient Consents

This study complied with the latest revision of the Declaration of Helsinki and was approved by the Ethics Committee of the Chinese PLA General Hospital (Approval No. S2021‐67701). All participants provided written informed consent for imaging and sampling procedures; where appropriate, ethical exemptions were granted.

### 2.2 Study Population

This study included 69 patients with CD and 64 healthy controls (HC), recruited from the Department of Neurosurgery at the First Medical Center of the Chinese PLA General Hospital. The inclusion criteria for patients with CD were as follows: (1) age between 18 and 65 years, (2) diagnosis confirmed in accordance with the “Consensus on Diagnosis and Management of Cushing's Disease: Guideline Update” (Fleseriu et al. 2021), (3) availability of complete clinical and laboratory data, (4) no administration of exogenous glucocorticoids within three months before MRI examination, and (5) absence of major neurological, psychiatric, or systemic comorbidities. HC participants were required to have no neurological or systemic diseases potentially affecting the central nervous system, normal neurological examination findings, and no history of glucocorticoid use.

### 2.3 Cortisol Measurements

Blood samples were collected from patients with CD at 8 a.m., 4 p.m., and 00:00. To further evaluate endogenous cortisol secretion in patients with CD, serum samples were additionally obtained from HC at 8 a.m. for cortisol measurement and comparison. Serum cortisol levels in patients with CD were determined using a chemiluminescence immunoassay.

### 2.4 MRI Acquisition

Whole‐brain imaging was performed on a 3.0 T scanner (Manufacturer, Model) with a 32‐channel head coil. The protocol included diffusion‐weighted imaging (DWI) and a 3D T1‐weighted structural sequence. Head motion was minimized using foam padding and immobilization straps.

Diffusion‐weighted images were acquired with a TR/TE of 7522/80.8 ms, a field of view (FOV) of 224 × 224 mm^2^, and a matrix size of 112 × 112, resulting in a 2‐mm isotropic voxel resolution. The sequence included 64 diffusion‐encoding directions with a *b*‐value of 1000 s/mm^2^ and four nondiffusion‐weighted volumes (*b* = 0).

Structural anatomical images were acquired using a 3D T1WI MPRAGE sequence (TR/TE = 2300/2.3 ms; TI = 900 ms; flip angle = 9°). This sequence covered a 256 × 256 mm^2^ FOV with a 256 × 256 matrix and 1.0 mm slice thickness, yielding 1‐mm isotropic resolution. All acquisition parameters were maintained consistently throughout the study to ensure data stability.

### 2.5 Processing of MRI

3D‐T1WI data preprocessing was performed using FSL. The preprocessing steps included DICOM‐to‐NIfTI conversion using dcm2niix, followed by brain extraction (skull stripping) with BET (fractional intensity threshold = 0.2) to generate a skull‐stripped T1‐weighted image and the corresponding brain mask. The outputs were used for subsequent analyses.

Processing of DWI data began with MP‐PCA denoising and Gibbs unringing. Eddy‐current and motion correction with outlier slice replacement were then performed using FSL's eddy tool, followed by N4 bias‐field correction. Brain extraction was carried out on the first *b* = 0 volume using FSL's BET to generate brain masks. Diffusion tensors were subsequently fitted by linear least‐squares regression on the non‐zero *b*‐value shells. Eigenvalues and eigenvectors were obtained through tensor diagonalization, and FA maps were derived from these components.

### 2.6 Calculation of the ALPS Index

**FIGURE 1:**
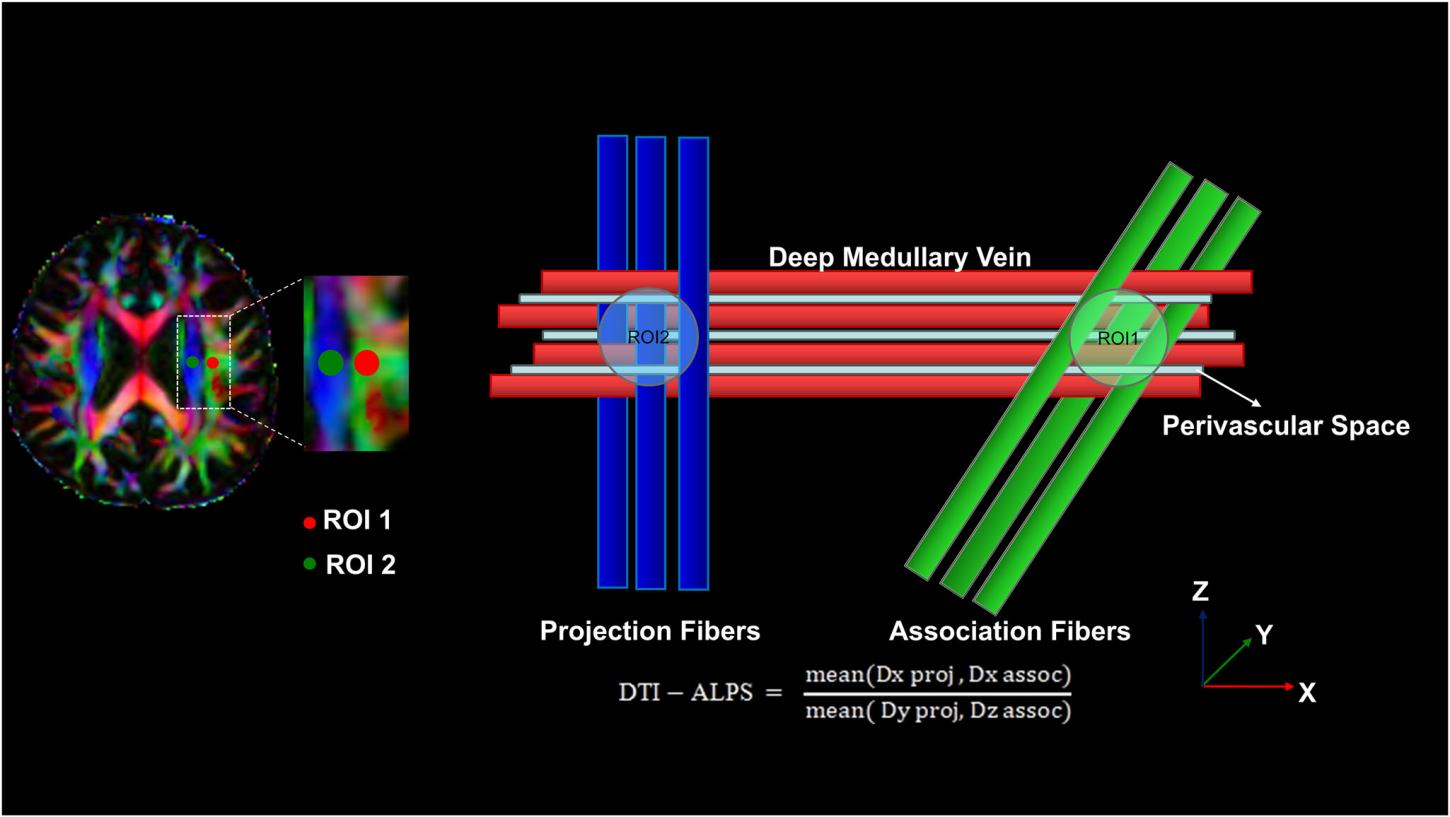
Representative ROIs for ALPS index calculation. Direction‐encoded color FA map at the lateral ventricle level (axial view) showing the association fiber ROI (green, association fibers) and projection fiber ROI (blue, projection fibers) positioned along the subventricular deep medullary veins.

Following DWI preprocessing, directionally encoded color (DEC) FA maps were generated using the standard RGB convention (red: left–right [x‐axis]; green: anterior–posterior [y‐axis]; blue: superior–inferior [z‐axis]). On axial slices at the level of the lateral ventricles, cubic regions of interest (ROIs, 3 × 3 × 3 mm^3^) were manually placed along the trajectory of the deep periventricular medullary veins. Specifically, one ROI was positioned within the association fibers (oriented along the y‐axis, appearing green) and another within the projection fibers (oriented along the z‐axis, appearing blue) (Figure 1). All ROIs were independently and blindly delineated by a neurosurgeon and a neuroradiologist, with excellent inter‐rater reliability (intraclass correlation coefficient [ICC] = 0.82). For each rater, the apparent diffusion coefficients (Dx, Dy, Dz) along the x, y, and z directions were extracted from each ROI, and the ALPS index was calculated using the following formula (Taoka et al. 2017):

$$\mathrm{DTI}-\mathrm{ALPS}\;=\frac{\mathrm{mean}\left(\mathrm{Dx}\;\mathrm{proj},\mathrm{Dx}\;\mathrm{assoc}\right)}{\mathrm{mean}\left(\mathrm{Dy}\;\mathrm{proj},\;\mathrm{Dz}\;\mathrm{assoc}\right)},$$

where Dx proj and Dx assoc represent the MD along the x‐axis in the projection and association fiber regions, respectively; Dy proj represents the MD along the y‐axis in the projection fiber region; and Dz assoc represents the MD along the z‐axis in the association fiber region. Given prior evidence demonstrating high inter‐hemispheric concordance for ALPS values, alongside superior reproducibility and stability of ROI placement in the left hemisphere (Taoka et al. 2022), we utilized the mean ALPS index derived from three consecutive slices in the left hemisphere as the representative metric of glymphatic function for each participant.

### 2.7 Calculation of WM Tract Metrics

**FIGURE 2:**
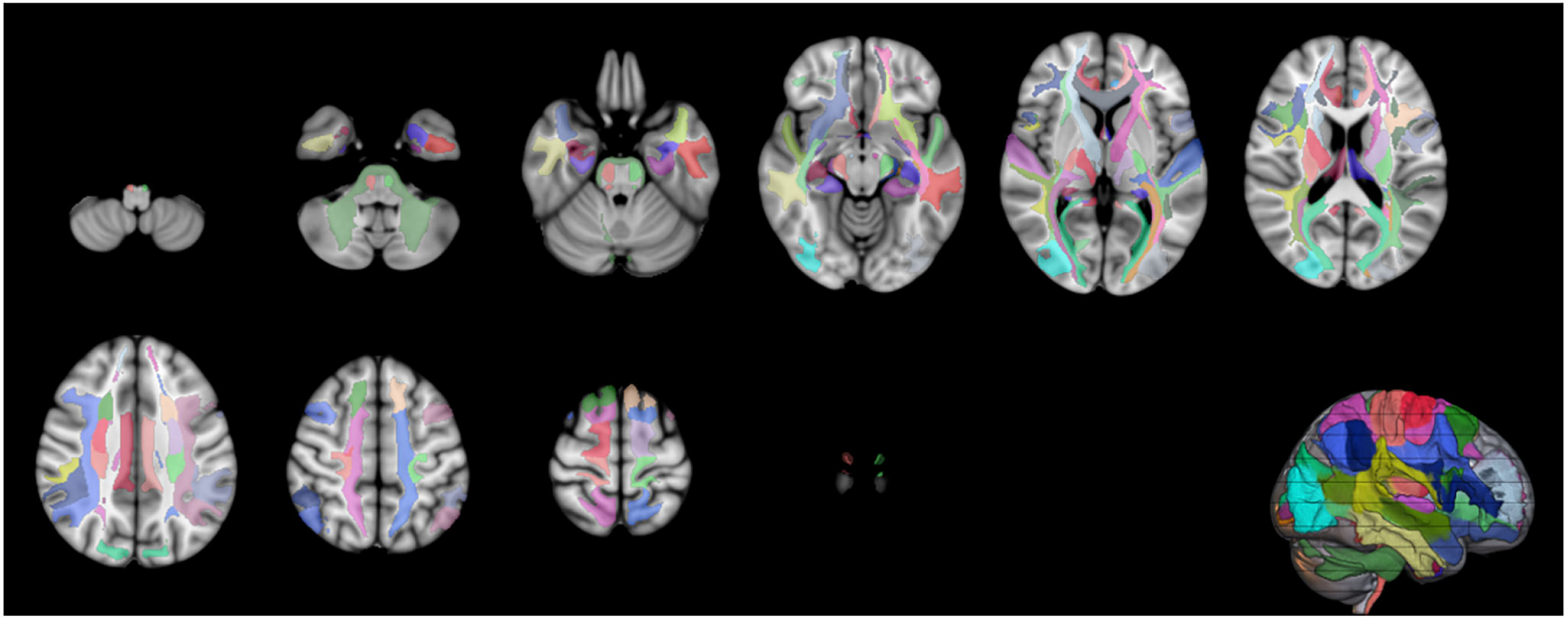
3D reconstruction of whole‐brain WM tracts using the XTRACT framework. The figure illustrates a 3D rendering of 42 major WM tracts in a representative subject (for a detailed list of all reconstructed WM tracts, please refer to Table S1). All tracts were automatically extracted using the standardized anatomical protocols implemented in the FSL‐XTRACT toolbox.

After DWI preprocessing and tensor fitting, we constructed a probabilistic fiber‐orientation model using BedpostX from the FSL toolbox, allowing up to two fiber populations per voxel (*n* = 2). The FA map, skull‐stripped T1‐weighted structural image, and standard MNI152 template were then linearly and nonlinearly registered with FLIRT and FNIRT, yielding bidirectional transformation matrices between diffusion space and standard space (Figure 2). Using these transformations, Xtract (HUMAN template) was applied to automatically segment 42 major WM fiber bundles in each participant's native diffusion space (Warrington et al. 2020). The xtract_stats utility was subsequently used to compute FA and MD for each bundle.

### 2.8 Statistical Analysis

All statistical analyses were conducted in R (version 4.4.2). Group differences in demographic and clinical variables were evaluated using independent‐samples *t* tests for normally distributed continuous data, Mann–Whitney *U* tests for non‐normally distributed continuous data, and χ^2^ tests for categorical variables. All tests were two‐tailed, and *p* < 0.05 was considered statistically significant.

Between‐group differences in the DTI‐ALPS index and WM tract metrics were assessed using linear models while adjusting for age, sex, ED, BMI, and total brain volume (TBV). Pearson's correlation analyses and a simple mediation model were subsequently performed to examine the associations among cortisol levels, the DTI‐ALPS index, and WM tract measures, as well as the potential mediating role of glymphatic function. We applied false discovery rate (FDR) correction for multiple comparisons.

## 3 Results

### 3.1 Demographic, Clinical, and Conventional MRI Variables in Patients With CD Compared With HC

A total of 69 patients diagnosed with CD were included in the study, comprising 6 males (8.7%) and 60 females (91.3%), with a mean age of 40.94 years. Additionally, 64 HCs were enrolled, consisting of 5 males (7.8%) and 59 females (92.2%), with a mean age of 41.94 years. The demographic, clinical, and MRI characteristics of both groups are summarized in Table 1. There were no statistically significant differences between the groups regarding age, sex, or education duration (ED) (all *p* > 0.05). However, compared with HC, patients with CD exhibited significantly higher 8 a.m. cortisol levels and BMI (all *p* < 0.05). In contrast, TBV was significantly lower in the CD group (*p* < 0.05).

**TABLE 1:** The demographic, clinical, and MRI characteristics of both groups.

Variable	CD(*n* = 69)	HC(*n* = 64)	*p*
Sex			0.855
Female	60	59	
Male	6	5	
Age	40.94 ± 10.99	41.91 ± 12.87	0.644
ED(year)	11.78 ± 4.28	10.83 ± 3.62	0.166
BMI(kg/m^2^)	26.32 ± 4.08	22.78 ± 3.33	< 0.001
8 a.m. Cortisol (nmol/L)	683.36 (524.74, 869.58)	330.36 (268.21, 411.30)	< 0.001
4 p.m. Cortisol (nmol/L)	604.28 (482.73,751.93)	—	—
00:00 Cortisol (nmol/L)	549.46 ± 221.59	—	—
TBV (mm^3^)	1,321,560.44 ± 116,852.65	1,384,686.91 ± 94,080.69	< 0.001
DTI‐ALPS	1.69 ± 0.20	1.76 ± 0.17	0.026

### 3.2 Between‐Group Differences in the DTI‐ALPS Index

**FIGURE 3:**
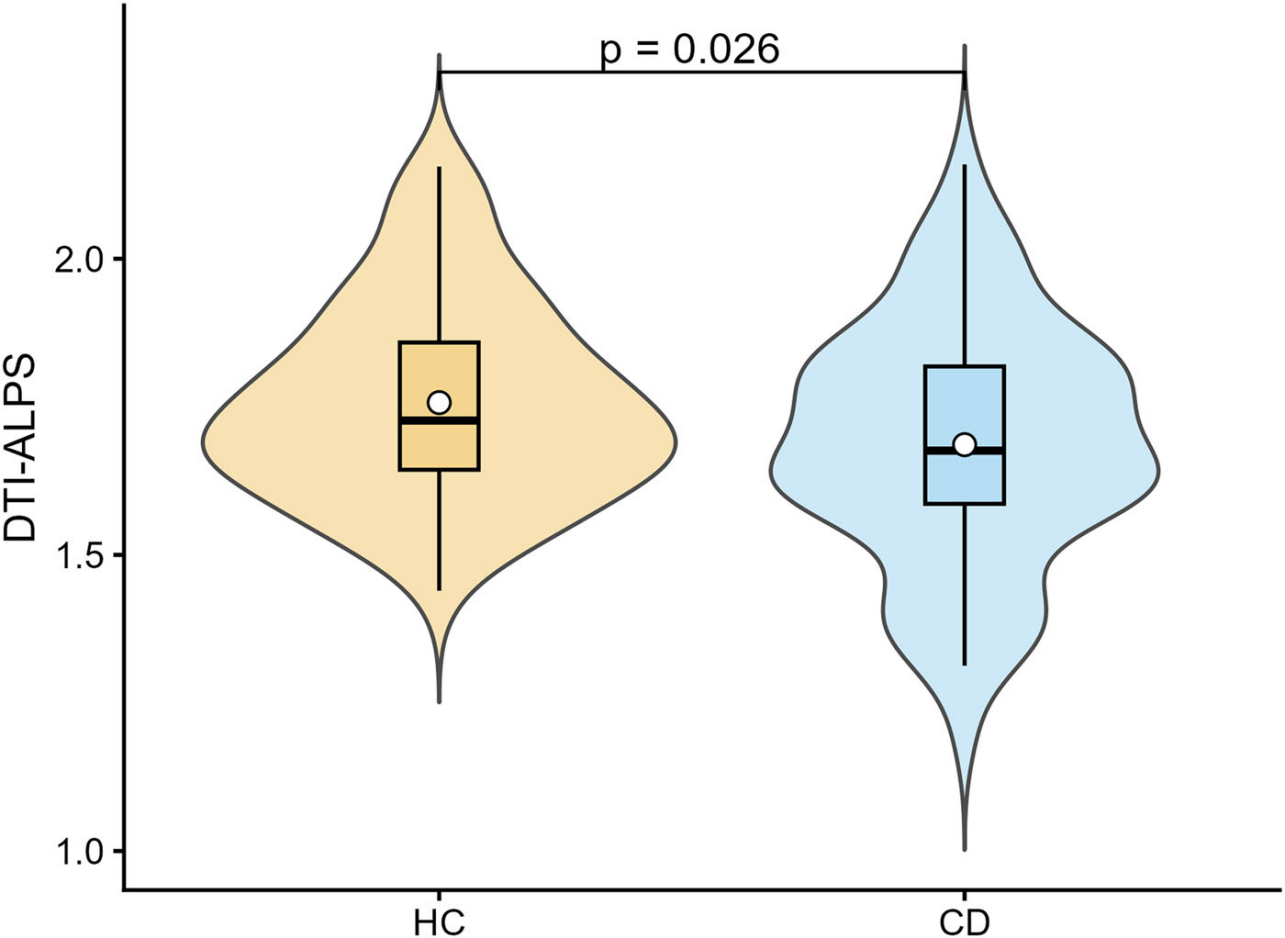
Comparison of the DTI‐ALPS index between the CD group and HC. Patients with CD exhibited significantly lower DTI‐ALPS indices compared to HC (*p* = 0.026).

After adjusting for age, sex, ED, BMI, and TBV, patients with CD exhibited a significantly lower DTI‐ALPS index compared to HC (CD: 1.69 ± 0.20 vs. HC:1.76 ± 0.17, FDR‐corrected *p* = 0.026; Figure 3, Table 1).

### 3.3 Between‐Group Differences in WM

**FIGURE 4:**
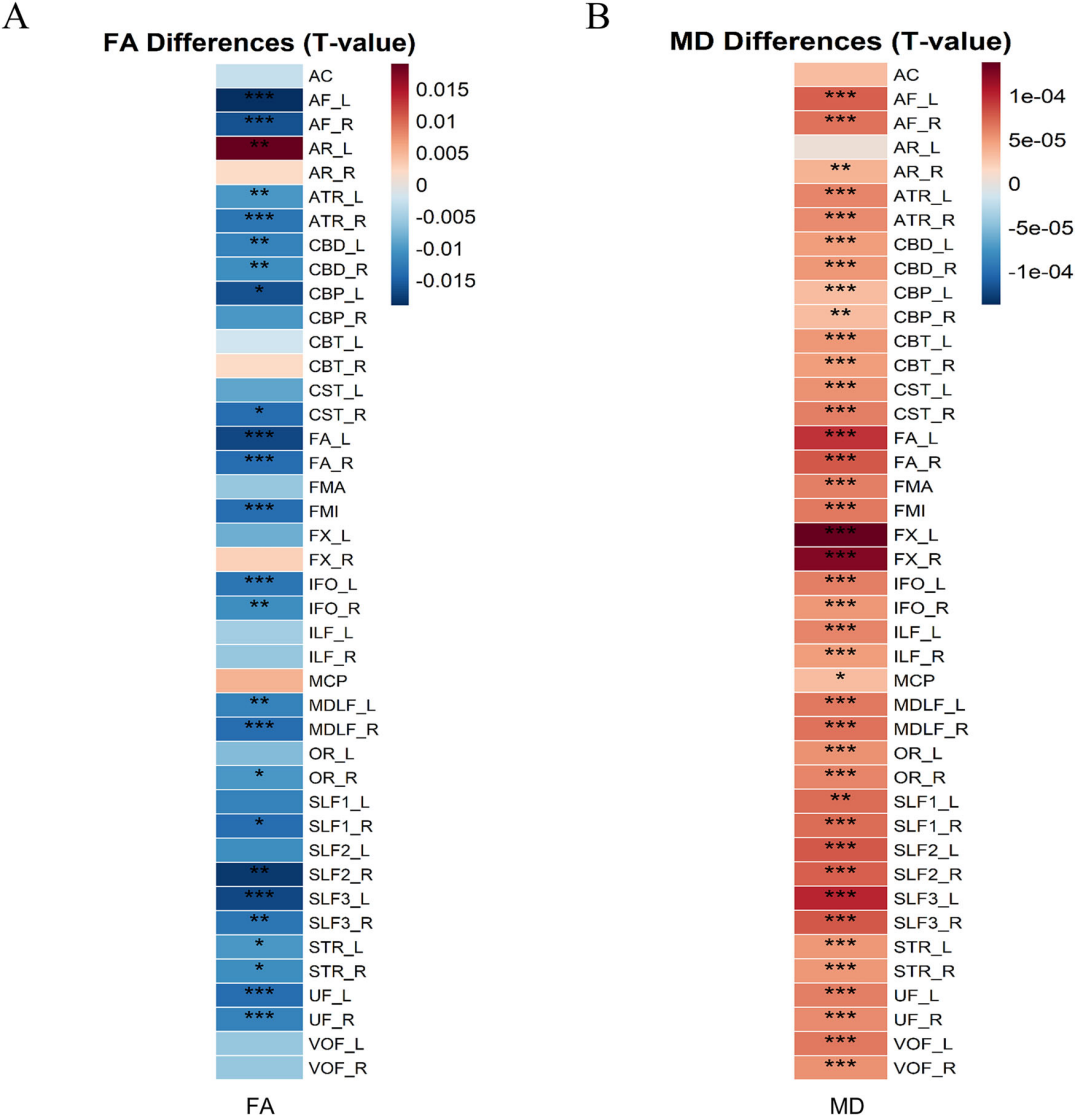
Heatmaps of microstructural differences across 42 WM tracts between the CD group and HC. (A) Distribution of T‐statistics for group differences in FA. (B) Distribution of T‐statistics for group differences in MD. Asterisks denote statistical significance levels: *p < 0.05, **p < 0.01, and ***p < 0.001.

After adjusting for age, sex, ED, BMI, and TBV, comparisons revealed widespread WM damage in patients with CD. FA values were significantly decreased in 25 WM tracts in the CD group compared to HC (FDR‐corrected *p* < 0.05; Figure 4A). In parallel, MD values showed a widespread increase in the CD group, affecting 40 of the 42 tracts (FDR‐corrected *p* < 0.05; Figure 4B).

### 3.4 Associations Between the DTI‐ALPS Index and WM Tract Injury Metrics in CD

**FIGURE 5:**
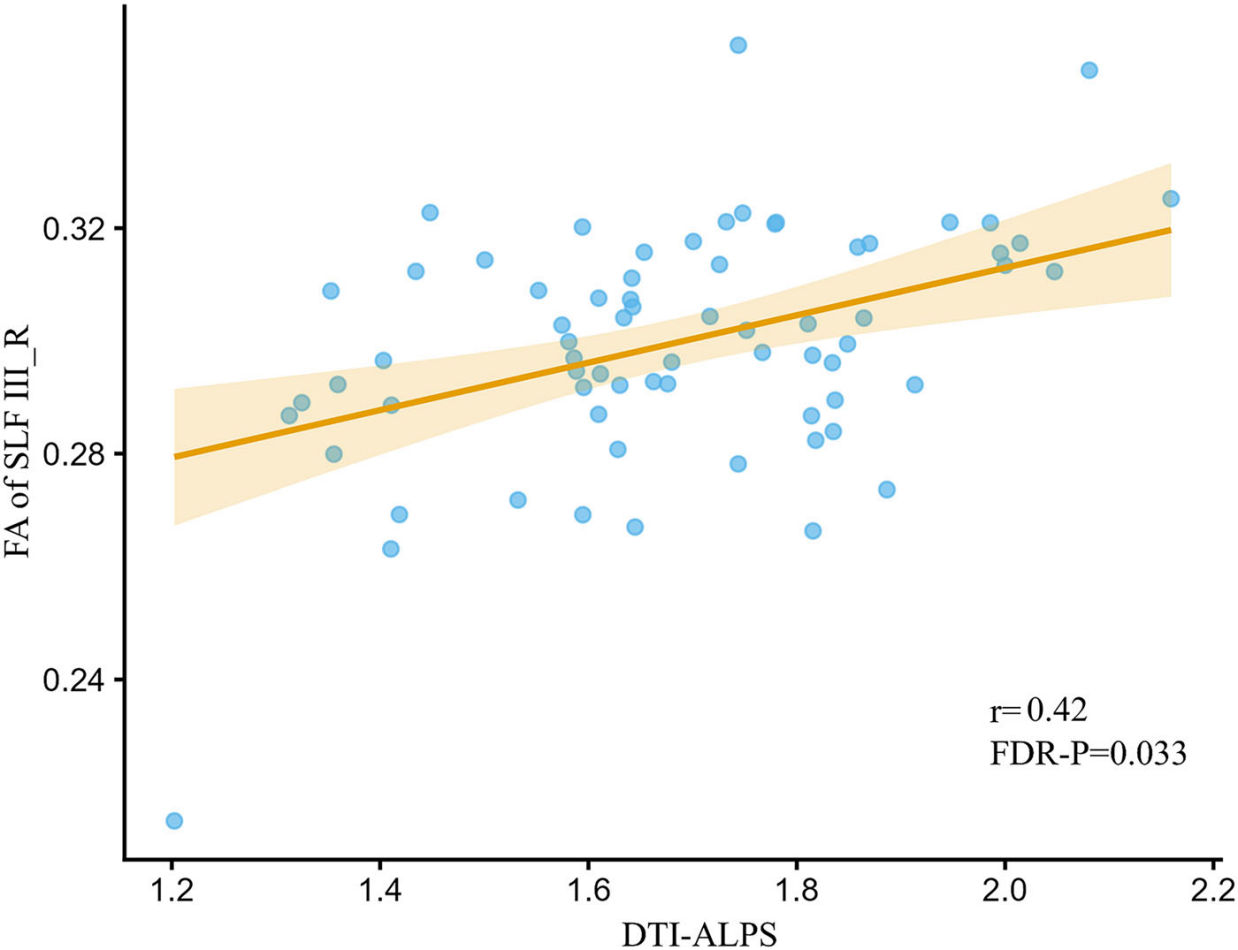
Associations between DTI‐ALPS and FA of SLF in SLF III_R individuals with CD. Pearson's correlation test indicated a positive correlation between DTI‐ALPS and FA of SLF III_R (*r* = 0.42; FDR‐ *p* = 0.033).

After adjusting for age, sex, ED, BMI, TBV, and cortisol levels at 00:00, 8 a.m., and 4 p.m., the DTI‐ALPS index was positively correlated with FA in the right SLF III (SLF III_R; *r* = 0.42, FDR‐corrected *p* = 0.033; Figure 5). No significant associations were detected between the DTI‐ALPS index and the remaining tracts that had shown between‐group alterations after FDR correction.

### 3.5 Associations Between Cortisol Levels and the DTI‐ALPS Index

**FIGURE 6:**
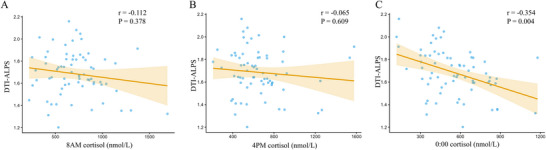
Associations between cortisol levels and DTI‐ALPS individuals with CD. (A) Pearson's correlation analysis between 8 a.m. cortisol levels and DTI‐ALPS; (B) Pearson's correlation analysis between 4 p.m. cortisol levels and DTI‐ALPS; (C) Pearson's correlation test indicated a negative correlation between 00:00 cortisol and DTI‐ALPS (*r* = −0.354; *p* = 0.004).

We further investigated the relationship between cortisol levels and DTI‐ALPS index in patients with CD. After adjusting for age, sex, ED, BMI, and TBV, the analysis revealed a significant negative correlation between 00:00 cortisol levels and the DTI‐ALPS index (*r* = −0.354, *p* = 0.004; Figure 6C). In contrast, no significant correlations were observed between the DTI‐ALPS index and 8 a.m. or 4 p.m. cortisol levels (all *p* > 0.05; Figure 6A and B).

### 3.6 Mediation Analysis: DTI‐ALPS Index Mediates the Effect of 00:00 Cortisol Levels on FA of SLF III_R in CD

**FIGURE 7:**
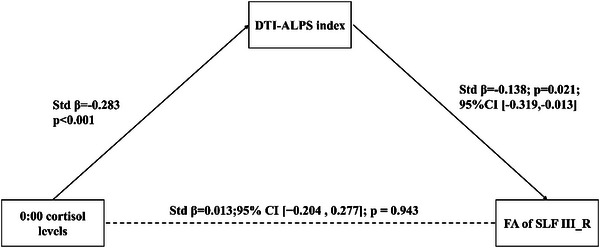
DTI‐ALPS index mediates the effect of 00:00 cortisol levels on FA of SLF III_R in CD.

Mediation analysis indicated that the DTI‐ALPS index mediated the association between 00:00 cortisol level and the FA value of SLF III_R (ACME = −0.138, 95% CI: [−0.319, −0.013], *p* = 0.021; ADE = 0.013, 95% CI [−0.204, 0.277], *p* = 0.943; TE = −0.126, 95% CI [−0.285, 0.055], *p* = 0.162), Models demonstrated a full mediation effect. These findings suggest that the association between 00:00 cortisol and the alterations in FA of SLF III_R is primarily mediated through glymphatic function (Figure 7).

## 4 Discussion

This study evaluated glymphatic function and WM tract characteristics in patients with CD. The main findings can be summarized as follows. First, compared with HC, patients with CD exhibited impaired glymphatic function, as evidenced by a reduced DTI‐ALPS index, along with widespread WM microstructural alterations. Second, impaired glymphatic function was significantly associated with 00:00 cortisol levels in CD. Third, mediation analysis demonstrated that glymphatic impairment fully mediated the relationship between 00:00 cortisol levels and decreased FA in the SLF III_R.

The glymphatic system is regarded as a critical pathway for the clearance of interstitial solutes and metabolic waste within the central nervous system (Iliff et al. 2012). Previous studies have indicated that a decline in glymphatic clearance efficiency is associated with impaired solute removal and enhanced inflammatory responses in the brain (Iliff et al. 2012; Mogensen et al. 2021; Jessen et al. 2015). Furthermore, these alterations have been linked to abnormalities in WM microstructure (Sabayan and Westendorp 2021; Carotenuto et al. 2022), which may provide a mechanistic framework to interpret the findings of the present study. Notably, this study suggests that impaired glymphatic function in CD patients may predominantly involve the SLF III. The SLF III primarily connects the inferior parietal lobule with the inferior frontal gyrus and adjacent language‐related regions (Makris et al. 2005). It constitutes a critical fiber tract within the frontoparietal network involved in phonological processing and sensorimotor integration (Latini et al. 2020). Given that intense neural activity and associated energy metabolism typically entail increased production of solutes and heightened demands for homeostatic regulation (Attwell and Laughlin 2001; Raichle and Gusnard 2002), the SLF III may exhibit greater vulnerability under conditions of impaired clearance efficiency. Consistent with this, Qiu et al. (2025) reported a significant correlation between glymphatic functional impairment and abnormalities in diffusion metrics of the SLF, specifically decreased FA and increased MD, in children with refractory epilepsy. This finding suggests that this pattern may exhibit a degree of cross‐disease consistency; however, its comparability requires further examination in the context of disease mechanisms and population differences.

Previous studies have indicated that elevated levels of cortisol may be associated with the disruption of the polarized distribution of astrocytic aquaporin‐4 (AQP4), thereby reducing the efficiency of cerebrospinal fluid–interstitial fluid (CSF‐ISF) exchange (Wei et al. 2019). This mechanistic insight provides a conceptual framework for understanding impaired glymphatic function. Within this context, abnormally increased cortisol levels may represent a potential contributing factor to the decline of glymphatic function observed in patients with CD. Concurrently, previous studies on major depressive disorder have also reported a negative correlation between cortisol levels and glymphatic function (Chen et al. 2025), thereby providing cross‐population evidence supporting the association pattern of elevated cortisol accompanied by reduced glymphatic function. This study further identified a significant negative correlation between 00:00 cortisol levels and impaired glymphatic function, as characterized by DTI‐ALPS, in patients with CD. Given that the glymphatic system exhibits a pronounced circadian rhythm (Ringstad 2024; Hablitz et al. 2020), with nocturnal periods typically representing a relatively active phase for metabolic waste clearance (Hablitz et al. 2020; Xie et al. 2013), these findings suggest that 00:00 cortisol levels may adversely affect glymphatic function by attenuating the nocturnal clearance peak or disrupting the underlying rhythmic processes.

Mediation analysis revealed that glymphatic function significantly mediates the relationship between cortisol levels at 00:00 and microstructural abnormalities in the SLF III_R. This finding suggests that the association between nocturnal cortisol dysregulation and specific WM tract impairment may be partially attributable to impaired glymphatic function. Previous studies have predominantly attributed WM abnormalities in patients with CD to cortisol‐associated neurotoxicity (van der Werff et al. 2014; Chen et al. 2020); however, the mechanism of direct neurotoxicity alone is insufficient to fully explain the spatially selective patterns of WM involvement observed in CD (Cui et al. 2021; Chen et al. 2020). From the perspective of the glymphatic function, we propose that the interplay between regional metabolic demands and variations in glymphatic function may serve as a critical complementary mechanism underlying this tract‐specific vulnerability, thereby extending current understanding of WM damage in CD.

To our knowledge, this study is the first to identify glymphatic dysfunction in patients with CD and to reveal that this dysfunction leads to damage in specific WM tracts. This study offers a novel perspective on the mechanisms underlying WM tract damage in patients with CD. Furthermore, the research findings suggest that controlling nighttime cortisol levels may help alleviate glymphatic dysfunction, thereby mitigating damage to certain WM tracts.

Our study has several limitations, warranting caution in interpreting the results. First, the cross‐sectional design of this study limits the assessment of causal relationships and cannot track the dynamic changes in glymphatic function and WM damage over time. Future longitudinal studies are needed to clarify the temporal evolution of these changes following disease remission. Second, although the DTI‐ALPS index serves as a valuable noninvasive surrogate marker for assessing glymphatic function, it specifically quantifies diffusion capacity along the perivascular spaces of medullary veins. Given that the glymphatic system encompasses complex fluid dynamics extending beyond localized perivascular diffusion, this index may not fully capture the global characteristics of glymphatic function throughout the brain. To achieve a more comprehensive evaluation of the glymphatic‐interstitial fluid system, future research should adopt multimodal approaches that integrate additional relevant imaging biomarkers, such as perivascular space (PVS) burden, choroid plexus (CP) volume, and free water (FW) imaging, for combined analysis. Third, sleep disturbances are common comorbidities in patients with CD and are recognized as significant modulators of glymphatic function. This analysis did not explicitly control for the potential confounding effect of sleep quality. Future research should incorporate comprehensive assessments of sleep in order to delineate the specific contributions of hypercortisolemia and sleep disorders to lymphatic dysfunction. Finally, although the DTI‐ALPS index has been widely used as a surrogate marker of glymphatic clearance, its signal sources and biological specificity remain debated; therefore, interpretation should be made with caution.

In summary, our study identifies significant glymphatic functional impairment in patients with CD. Our findings suggest that aberrantly elevated 00:00 cortisol levels serve as a primary driver of glymphatic functional impairment. Furthermore, impaired glymphatic function plays a critical role in mediating microstructural injury to the SLF III_R. Collectively, these results provide novel insights for a better understanding of WM damage in patients with CD.

## Author Contributions

Y.S. and Y.Z. conceptualized the study. Y.S., J.X., and Q.W. were responsible for the methodology. Y.S., H.L., and Z.Z. performed the software analysis and formal analysis. J.X., H.L., and X.Y. conducted the investigation and data curation. Y.S. and Z.Z. were responsible for visualization. Y.S. wrote the original draft. X.Y. and Y.Z. reviewed and edited the manuscript. Y.Z. and X.Y. were responsible for supervision and funding acquisition. All authors have read and approved the final manuscript.

## Funding

This work was supported by the National Science and Technology Major Project of the Ministry of Science and Technology of China (No. 2022ZD0210100) and the Young Talent Project of Chinese PLA General Hospital (Grant Nos. 20230403 to Yanyang Zhang).

## Ethics Statement

This study was approved by the Ethics Committee of the Chinese PLA General Hospital (Approval No. [S2021‐67701]). All participants provided written informed consent prior to inclusion in the study.

## Conflicts of Interest

The authors declare no conflicts of interest.

## Supporting information




**Supplementary Table**: brb371285‐sup‐0001‐tableS1.docx

## Data Availability

Data are available from the corresponding authors on reasonable request.

## References

[brb371285-bib-0010] Alexander, A. L. , J. E. Lee , M. Lazar , and A. S. Field . 2007. “Diffusion Tensor Imaging of the Brain.” Neurotherapeutics 4, no. 3: 316–329. 17599699 10.1016/j.nurt.2007.05.011 PMC2041910

[brb371285-bib-0023] Attwell, D. , and S. B. Laughlin . 2001. “An Energy Budget for Signaling in the Grey Matter of the Brain.” Journal of Cerebral Blood Flow and Metabolism 21, no. 10: 1133–1145. 11598490 10.1097/00004647-200110000-00001

[brb371285-bib-0011] Budde, M. D. , M. Xie , A. H. Cross , and S. K. Song . 2009. “Axial Diffusivity is the Primary Correlate of Axonal Injury in the Experimental Autoimmune Encephalomyelitis Spinal Cord: A Quantitative Pixelwise Analysis.” Journal of Neuroscience 29, no. 9: 2805–2813. 19261876 10.1523/JNEUROSCI.4605-08.2009 PMC2673458

[brb371285-bib-0015] Carlstrom, L. P. , A. Eltanahy , A. Perry , et al. 2022. “A Clinical Primer for the Glymphatic System.” Brain 145, no. 3: 843–857. 34888633 10.1093/brain/awab428

[brb371285-bib-0014] Carotenuto, A. , L. Cacciaguerra , E. Pagani , P. Preziosa , M. Filippi , and M. A. Rocca . 2022. “Glymphatic System Impairment in Multiple Sclerosis: Relation With Brain Damage and Disability.” Brain 145, no. 8: 2785–2795. 34919648 10.1093/brain/awab454

[brb371285-bib-0005] Chen, Y. , J. Zhang , H. Tan , J. Li , and Y. Yu . 2020. “Detrimental Effects of Hypercortisolism on Brain Structure and Related Risk Factors.” Scientific Reports 10, no. 1: 12708. 32728036 10.1038/s41598-020-68166-0 PMC7391644

[brb371285-bib-0027] Chen, S. , Z. Xu , Z. Guo , et al. 2025. “Glymphatic Dysfunction Associated With Cortisol Dysregulation in Major Depressive Disorder.” Translation Psychiatry 15, no. 1: 265. 10.1038/s41398-025-03486-1 PMC12325902 40764475

[brb371285-bib-0003] Cui, M. , T. Zhou , S. Feng , et al. 2021. “Altered Microstructural Pattern of White Matter in Cushing's Disease Identified by Automated Fiber Quantification.” NeuroImage Clinical 31: 102770. 34332193 10.1016/j.nicl.2021.102770 PMC8339293

[brb371285-bib-0001] Fleseriu, M. , R. Auchus , I. Bancos , et al. 2021. “Consensus on Diagnosis and Management of Cushing's Disease: A Guideline Update.” Lancet Diabetes & Endocrinology 9, no. 12: 847–875. 34687601 10.1016/S2213-8587(21)00235-7 PMC8743006

[brb371285-bib-0029] Hablitz, L. M. , V. Plá , M. Giannetto , et al. 2020. “Circadian Control of Brain Glymphatic and Lymphatic Fluid Flow.” Nature Communications 11, no. 1: 4411. 10.1038/s41467-020-18115-2 PMC7468152 32879313

[brb371285-bib-0018] Iliff, J. J. , M. Wang , Y. Liao , et al. 2012. “A Paravascular Pathway Facilitates CSF Flow Through the Brain Parenchyma and the Clearance of Interstitial Solutes, Including Amyloid β.” Science Translational Medicine 4, no. 147: 147ra111. 10.1126/scitranslmed.3003748 PMC3551275 22896675

[brb371285-bib-0020] Jessen, N. A. , A. S. Munk , I. Lundgaard , and M. Nedergaard . 2015. “The Glymphatic System: A Beginner's Guide.” Neurochemical Research 40, no. 12: 2583–2599. 25947369 10.1007/s11064-015-1581-6 PMC4636982

[brb371285-bib-0012] Kuwahara, S. , M. Kawada , and S. Uga . 2001. “Chronic Subdural Hematoma With Vasogenic Edema in the Cerebral Hemisphere–Case Report.” Neurologia Medico‐Chirurgica 41, no. 4: 196–200. 11381678 10.2176/nmc.41.196

[brb371285-bib-0022] Latini, F. , G. Trevisi , M. Fahlström , et al. 2020. “New Insights Into the Anatomy, Connectivity and Clinical Implications of the Middle Longitudinal Fasciculus.” Frontiers in Neuroanatomy 14: 610324. 33584207 10.3389/fnana.2020.610324 PMC7878690

[brb371285-bib-0021] Makris, N. , D. N. Kennedy , S. McInerney , et al. 2005. “Segmentation of Subcomponents Within the Superior Longitudinal Fascicle in Humans: A Quantitative, In Vivo, DT‐MRI Study.” Cerebral Cortex 15, no. 6: 854–869. 15590909 10.1093/cercor/bhh186

[brb371285-bib-0019] Mogensen, F. L. , C. Delle , and M. Nedergaard . 2021. “The Glymphatic System (En)During Inflammation.” International Journal of Molecular Sciences 22, no. 14: 1791. 34299111 10.3390/ijms22147491 PMC8305763

[brb371285-bib-0009] Park, C. J. , S. Y. Kim , J. H. Kim , et al. 2023. “Evaluation of Glymphatic System Activity Using Diffusion Tensor Image Analysis Along the Perivascular Space and Amyloid PET in Older Adults With Objectively Normal Cognition: A Preliminary Study.” Frontiers in Aging Neuroscience 15: 1221667. 37577357 10.3389/fnagi.2023.1221667 PMC10413261

[brb371285-bib-0013] Qin, Y. , X. Li , Y. Qiao , et al. 2023. “DTI‐ALPS: An MR Biomarker for Motor Dysfunction in Patients With Subacute Ischemic Stroke.” Frontiers in Neuroscience 17: 1132393. 37065921 10.3389/fnins.2023.1132393 PMC10102345

[brb371285-bib-0025] Qiu, L. , M. Wang , S. Liu , et al. 2025. “Multi‐Parameter MRI for Evaluating Glymphatic Impairment and White‐Matter Abnormalities and Discriminating Refractory Epilepsy in Children.” Korean Journal of Radiology 26, no. 5: 485–497. 40307202 10.3348/kjr.2024.0718 PMC12055269

[brb371285-bib-0024] Raichle, M. E. , and D. A. Gusnard . 2002. “Appraising the Brain's Energy Budget.” PNAS 99, no. 16: 10237–10239. 12149485 10.1073/pnas.172399499 PMC124895

[brb371285-bib-0028] Ringstad, G. 2024. “Glymphatic Imaging: A Critical Look at the DTI‐ALPS Index.” Neuroradiology 66, no. 2: 157–160. 38197950 10.1007/s00234-023-03270-2

[brb371285-bib-0007] Sabayan, B. , and R. Westendorp . 2021. “Neurovascular‐Glymphatic Dysfunction and White Matter Lesions.” Geroscience 43, no. 4: 1635–1642. 33851307 10.1007/s11357-021-00361-x PMC8492845

[brb371285-bib-0008] Taoka, T. , Y. Masutani , H. Kawai , et al. 2017. “Evaluation of Glymphatic System Activity With the Diffusion MR Technique: Diffusion Tensor Image Analysis Along the Perivascular Space (DTI‐ALPS) in Alzheimer's Disease Cases.” Japanese Journal of Radiology 35, no. 4: 172–178. 28197821 10.1007/s11604-017-0617-z

[brb371285-bib-0016] Taoka, T. , R. Ito , R. Nakamichi , et al. 2022. “Reproducibility of Diffusion Tensor Image Analysis Along the Perivascular Space (DTI‐ALPS) for Evaluating Interstitial Fluid Diffusivity and Glymphatic Function: CHanges in Alps Index on Multiple conditiON acquIsition eXperiment (CHAMONIX) Study.” Japanese Journal of Radiology 40, no. 2: 147–158. 34390452 10.1007/s11604-021-01187-5 PMC8803717

[brb371285-bib-0006] Wang, D. J. , J. Hua , D. Cao , and M. L. Ho . 2023. “Neurofluids and the Glymphatic System: Anatomy, Physiology, and Imaging.” Bjr 96, no. 1151: 20230016. 37191063 10.1259/bjr.20230016 PMC10607419

[brb371285-bib-0017] Warrington, S. , K. L. Bryant , A. A. Khrapitchev , et al. 2020. “XTRACT—Standardised Protocols for Automated Tractography in the Human and Macaque Brain.” Neuroimage 217: 116923. 32407993 10.1016/j.neuroimage.2020.116923 PMC7260058

[brb371285-bib-0026] Wei, F. , J. Song , C. Zhang , et al. 2019. “Chronic Stress Impairs the Aquaporin‐4‐Mediated Glymphatic Transport Through Glucocorticoid Signaling.” Psychopharmacology 236, no. 4: 1367–1384. 30607477 10.1007/s00213-018-5147-6

[brb371285-bib-0002] van der Werff, S. J. , C. D. Andela , J. Nienke Pannekoek , et al. 2014. “Widespread Reductions of White Matter Integrity in Patients With Long‐Term Remission of Cushing's Disease.” NeuroImage Clinical 4: 659–667. 24936417 10.1016/j.nicl.2014.01.017 PMC4053612

[brb371285-bib-0030] Xie, L. , H. Kang , Q. Xu , et al. 2013. “Sleep Drives Metabolite Clearance From the Adult Brain.” Science 342, no. 6156: 373–377. 24136970 10.1126/science.1241224 PMC3880190

[brb371285-bib-0004] Xu, C. X. , L. Kong , H. Jiang , et al. 2024. “Analysis of Brain Structural Covariance Network in Cushing Disease.” Heliyon 10, no. 7: e28957. 38601682 10.1016/j.heliyon.2024.e28957 PMC11004566

