# Supplementary material for: Association Between Glymphatic Function and White Matter Microstructural Injury in Patients With Cushing's Disease
Source: Brain Behav. 2026 Feb 28;16(3):e71285. doi: 10.1002/brb3.71285 (PMC12949719; doi:10.1002/brb3.71285)
Supplement: Supplementary file 1 — Supplementary Table: brb371285‐sup‐0001‐tableS1.docx [file BRB3-16-e71285-s001.docx]

Supplementary Table 1:3D renderings of the 42 major WM tracts from XTRACT, along with their abbreviations.

|  | WM tract | Abbreviation |
| --- | --- | --- |
| 1 | Anterior Commissure | AC |
| 2 | Left Arcuate Fasciculus | AF_L |
| 3 | Right Arcuate Fasciculus | AF_R |
| 4 | Left Acoustic Radiation | AR_L |
| 5 | Right Acoustic Radiation | AR_R |
| 6 | Left Anterior Thalamic Radiation | ATR_L |
| 7 | Right Anterior Thalamic Radiation | ATR_R |
| 8 | Left Cingulum (Dorsal) | CBD_L |
| 9 | Right Cingulum (Dorsal) | CBD_R |
| 10 | Left Cingulum (Peri-genual) | CBP_L |
| 11 | Right Cingulum (Peri-genual) | CBP_R |
| 12 | Left Cingulum (Temporal) | CBT_L |
| 13 | Right Cingulum (Temporal) | CBT_R |
| 14 | Left Corticospinal Tract | CST_L |
| 15 | Right Corticospinal Tract | CST_R |
| 16 | Left Frontal Aslant | FA_L |
| 17 | Right Frontal Aslant | FA_R |
| 18 | Forceps Major | FMA |
| 19 | Forceps Minor | FMI |
| 20 | Left Fornix | FX_L |
| 21 | Right Fornix | FX_R |
| 22 | Left Inferior Fronto-Occipital Fasciculus | IFO_L |
| 23 | Right Inferior Fronto-Occipital Fasciculus | IFO_R |
| 24 | Left Inferior Longitudinal Fasciculus | ILF_L |
| 25 | Right Inferior Longitudinal Fasciculus | ILF_R |
| 26 | Middle Cerebellar Peduncle | MCP |
| 27 | Left Middle Longitudinal Fasciculus | MDLF_L |
| 28 | Right Middle Longitudinal Fasciculus | MDLF_R |
| 29 | Left Optic Radiation | OR_L |
| 30 | Right Optic Radiation | OR_R |
| 31 | Left Superior Longitudinal Fasciculus 1 | SLF1_L |
| 32 | Right Superior Longitudinal Fasciculus 1 | SLF I_R |
| 33 | Left Superior Longitudinal Fasciculus 2 | SLF II_L |
| 34 | Right Superior Longitudinal Fasciculus 2 | SLF II_R |
| 35 | Left Superior Longitudinal Fasciculus 3 | SLF III_L |
| 36 | Right Superior Longitudinal Fasciculus 3 | SLF III_R |
| 37 | Left Superior Thalamic Radiation | STR_L |
| 38 | Right Superior Thalamic Radiation | STR_R |
| 39 | Left Uncinate Fasciculus | UF_L |
| 40 | Right Uncinate Fasciculus | UF_R |
| 41 | Left Vertical Occipital Fasciculus | VOF_L |
| 42 | Right Vertical Occipital Fasciculus | VOF_R |
